# Genomic analysis and pneumococcal population dynamics across PCV implementation in South Korea, 1997–2023

**DOI:** 10.1099/mgen.0.001433

**Published:** 2025-07-17

**Authors:** Jeong-Ih Shin, Sung-Yeon Cho, Jiyon Chu, Chulmin Park, Minho Lee, Joon Young Song, Seung-Hyun Jung, Dong-Gun Lee

**Affiliations:** 1 Department of Medical Sciences, Graduate School of The Catholic University of Korea, Seoul, Republic of Korea; 2 Catholic Research Institute for Human Genome Polymorphism, College of Medicine, The Catholic University of Korea, Seoul, Republic of Korea; 3 Vaccine Bio Research Institute, College of Medicine, The Catholic University of Korea, Seoul, Republic of Korea; 4 Department of Internal Medicine, Division of Infectious Diseases, College of Medicine, The Catholic University of Korea, Seoul, Republic of Korea; 5 Catholic Hematology Hospital, Seoul St. Mary’s Hospital, Seoul, Republic of Korea; 6 Department of Life Science, Dongguk University-Seoul, Seoul, Republic of Korea; 7 Department of Internal Medicine, Division of Infectious Diseases, Korea University Guro Hospital, Korea University College of Medicine, Seoul, Republic of Korea; 8 Department of Biochemistry, College of Medicine, The Catholic University of Korea, Seoul, Republic of Korea

**Keywords:** antimicrobial resistance, global pneumococcal sequence cluster, pneumococcal conjugate vaccine, *Streptococcus pneumoniae*, whole-genome sequencing

## Abstract

*Streptococcus pneumoniae*, a clinically significant pathogen, causes invasive diseases in children and older adults. Pneumococcal conjugate vaccines (PCVs) have substantially reduced the incidence of vaccine serotype (VT) pneumococcal diseases. However, serotype replacement, characterized by the emergence of non-vaccine serotypes (NVTs), presents a persistent challenge to disease prevention. To address this, we analysed 236 pneumococcal isolates collected in South Korea between 1997 and 2023, spanning both pre- and post-PCV13 periods. Whole-genome sequencing was performed to assess serotypes, antimicrobial resistance, virulence factors and global pneumococcal sequence clusters (GPSCs). Capsular switching events and the relationships among pneumococcal lineages, serotypes and disease invasiveness were also evaluated. Among the 37 identified serotypes, NVTs such as 23A, 15B/15C and 10A were dominant post-PCV13. Serotype 10A, associated with invasive pneumococcal diseases (IPDs), belonged to GPSC634-ST11189 and showed elevated minimum inhibitory concentrations for *β*-lactams. Capsular switching events were observed between VTs and NVTs, highlighting the adaptability of pneumococcal populations. Antimicrobial non-susceptibility was highest for azithromycin (82.7%), followed by tetracycline (76.5%) and co-trimoxazole (70.4%), with higher rates observed in the post-PCV13 period. Notably, amoxicillin (*P*=0.049) and meropenem (*P*=0.002) showed significant non-susceptibility in the post-PCV13 period. Virulence factors *pspA* and *pfbA* were associated with IPDs, while pilus islet PI-1-related genes were more frequent in non-invasive cases. These findings underscore the importance of genomic surveillance to monitor pneumococcal population dynamics and inform public health strategies. The inclusion of serotype 10A in the recently approved PCV20 offers promise for further reducing the global burden of IPDs, including in South Korea.

**Impact Statement**

As one of the few genomic epidemiology studies focused on pneumococcal isolates in South Korea, this study provides critical insights into changes in serotypes, lineages, antimicrobial resistance and invasiveness before and after PCV13 introduction. Based on whole-genome sequencing data from 236 isolates collected over 27 years (1997–2023), we identified significant changes in the pneumococcal population structure and antimicrobial resistance patterns. Our study revealed the continued emergence of non-vaccine serotypes, including serotype 10A-ST11189, which had previously been reported in South Korea between 2014 and 2019. Additionally, we identified global pneumococcal sequence clusters associated with increased antimicrobial resistance that could pose future public health challenges. Our findings provide valuable insights for pneumococcal vaccine development and antimicrobial resistance management in South Korea.

## Data Summary

Whole-genome sequences have been deposited in the Sequence Read Archive, and the accession numbers, along with the complete metadata for each isolate, are provided in Table S1. A phylogenetic snapshot of *Streptococcus pneumoniae* isolates from South Korea is accessible at https://microreact.org/project/spn-southkorea-1997-2023-fig2.

The authors confirm all supporting data, code and protocols have been provided within the article or through supplementary data files.

## Introduction

*Streptococcus pneumoniae* is a clinically important pathogen responsible for a wide spectrum of diseases, ranging from non-invasive conditions such as otitis media and sinusitis to severe invasive diseases, including pneumonia, meningitis and bacteraemia [1]. Pneumococcal infections primarily affect vulnerable groups, including children, older adults and individuals with pre-existing medical conditions or compromised immune systems. Vaccination efforts, particularly the advent of pneumococcal conjugate vaccines (PCVs), have significantly reduced the incidence of invasive pneumococcal diseases (IPDs) caused by vaccine serotypes (VTs) in both children and adults [2,4]. However, serotype replacement and vaccine escape phenomena remain ongoing challenges [5]. Despite active pneumococcal vaccination efforts targeting high-risk groups, a study conducted in South Korea between 2018 and 2021 found that *S. pneumoniae* accounted for 11.8% of hospitalized pneumococcal community-acquired pneumonia cases, making it the most common causative agent [6]. In South Korea, IPD has been a nationally notifiable infectious disease since 2014. The incidence rate peaked at 1.29 cases per 100,000 population in 2018, declined thereafter and gradually rose again to 0.66 in 2022 and 0.69 in 2023. As of 2022, the overall mortality rate of IPD was 14.1% [7]. The pneumococcal carriage rate in nasopharyngeal swabs collected from children ranged from 36.4 to 42.1%, highlighting the persistent and significant burden of pneumococcal disease in South Korea [8]. Understanding the pathogenesis, epidemiology and mechanisms of resistance in *S. pneumoniae* is crucial for developing effective preventive and therapeutic interventions.

In South Korea, PCV7 (serotypes 4, 6B, 9V, 14, 18C, 19F and 23F) was introduced in 2003 and later replaced by PCV10 (adding serotypes 1, 5 and 7F) and PCV13 (further adding serotypes 3, 6A and 19A) in 2010 [9]. PCV10 and PCV13 were incorporated into the childhood National Immunization Program in 2014 [7]. Following the introduction of these vaccines, serotype replacement has been observed globally, characterized by a decline in VTs and a rise in non-vaccine serotypes (NVTs) [10]. For example, after the introduction of PCV7, there was a significant decrease in VTs, accompanied by an increase in NVTs, particularly 19A, which emerged as a leading cause of IPD [11]. Similarly, the spread of penicillin-resistant NVT 24F has been reported worldwide since the introduction of PCV13 [12].

A similar trend has been observed in Korean studies, where NVT replacement has occurred, with emerging clones exhibiting notable multidrug resistance and genetic diversification, including capsular switching and recombination [13,16]. However, these studies do not provide sufficient genomic context regarding the emergence and decline of specific serotypes in South Korea, highlighting a data gap that warrants further investigation. In addition, despite the increasing use of whole-genome sequencing (WGS) for global pathogen surveillance, the distribution of pneumococcal lineages, such as global pneumococcal sequence clusters (GPSCs), and their associations with serotypes, IPD and antimicrobial resistance (AMR), remain understudied in South Korea [13,16].

To address these gaps, we analysed WGS data from 236 *S*. *pneumoniae* clinical isolates collected in South Korea. Our primary aim was to characterize pneumococcal lineages and their dynamics with serotypes, IPD and AMR, providing critical insights to support vaccine development and guide effective disease management strategies.

## Methods

### Bacterial isolates

The pneumococcal isolates were consecutively collected from three university hospitals (Seoul St. Mary’s Hospital, Eunpyeong St. Mary’s Hospital and Korea University Medical Center) and three nationwide bacterial biobanks in South Korea (National Culture Collection for Pathogens, GNUH-NCCP and Green Cross Laboratories). For sequencing analysis, a total of 236 *S*. *pneumoniae* clinical isolates, available from both invasive and non-invasive pneumococcal disease cases collected between 1997 and 2023, were selected. Infections in which *S. pneumoniae* was isolated from normally sterile sites, such as blood or cerebrospinal fluid, were defined as IPD, while isolates from other sources, including sputum, nasal aspirates, open wounds and ear discharge, were categorized as non-IPD. All stocks of each isolate were plated on agar plates containing 5% sheep blood and incubated overnight at 37 °C in 5% CO_2_. The study was approved by the Institutional Review Board of the College of Medicine, Catholic University of Korea (MC22SNSI0058), and received a waiver for informed consent due to the anonymous nature of the clinical isolates.

### Serotyping and antimicrobial susceptibility testing

Serotyping was performed using multiplex PCR according to the Centers for Disease Control and Prevention protocol (https://www.cdc.gov/streplab/pcr.html). The multiplex PCR serotyping results were compared with the serotypes predicted from the WGS data. Antimicrobial susceptibility testing (AST) was conducted for penicillin, amoxicillin, meropenem, cefotaxime, vancomycin, levofloxacin, clindamycin, azithromycin, tetracycline and trimethoprim-sulfamethoxazole (co-trimoxazole) using the broth microdilution method. *S. pneumoniae* ATCC 49619 was used as the control strain. Results were interpreted using the Clinical Laboratory Standards Institute guideline with non-meningitis cutoff values [17].

### WGS and *de novo* assembly

Genomic DNA was extracted using the QIAamp DNA Mini Kit (Qiagen, Hilden, Germany). Next-generation sequencing libraries were prepared using the TruSeq Nano DNA sample preparation kit (Illumina, San Diego, CA) and sequenced on the MiSeq system (Illumina). Adapters and low-quality reads were trimmed using Trimmomatic (version 0.39) [18], and reads from each isolate were assembled using SPAdes (version 4.1.0) [19]. The assembled contigs were evaluated using Quast (https://github.com/ablab/quast) and then scaffolded using MeDuSa (version 1.6) [20]. The resulting assemblies were annotated using Prokka (version 1.14.5) [21]. Raw sequencing reads were deposited in Sequence Read Archive under the accession number PRJNA1066038.

### Bioinformatic analysis

Sequencing data processing was performed according to a previously described protocol [22]. Species were identified using Kraken2 (version 2.1.2) with the standard Kraken2 database (accessed June 2022) [23], and serotype was predicted using SeroBA (version 1.0.2) [24]. Serotypes 15B and 15C were grouped together as 15B/15C due to their interconversion properties [25]. Multi-locus sequence types (MLSTs) were determined using the MLST tools (version 2.23.0; https://github.com/tseemann/mlst), with the allelic profiles of the seven housekeeping genes (*aroE*, *gdh*, *gki*, *recP*, *spi*, *xpt* and *ddl*) from the PubMLST database [26]. Clonal complexes (CCs) were assigned using the single-locus variant threshold as previously described [27]. GPSCs were assigned using PopPUNK (version 2.6.3) and the GPS database (version 9) [28]. Potential capsular switching was defined as the emergence of a serotype within the same GPSC after the introduction of PCV (post-PCV), with evidence of recombination in the *cps* locus using Gubbins (version 3.2.1) [29]. AMR gene profiles, including penicillin-binding protein (PBP) types, *ermB* and *tetM*, were determined using Pathogenwatch [29]. The concordance between *in silico* predicted susceptibility results from Pathogenwatch and AST results was evaluated for identical or same-class antimicrobials. Virulence factor acquisition was determined using VFanalyzer (release 5) in the virulence factor database [30]. Details of the genome-based molecular typing are provided in Table S1, available in the online Supplementary Material.

A core gene alignment for 236 pneumococcal genomes was performed using Roary (version 3.13.0) with default settings [31]. A maximum-likelihood phylogenetic tree based on core gene SNPs was constructed using RAxML (version 8.2.12) with 1,000 bootstraps and a generalized time-reversible gamma model [32]. *S. pneumoniae* ATCC700669 (GenBank accession: FM211187) was used as an outgroup to root the tree. The resulting phylogenetic tree was visualized in Microreact [33]. For the global comparative analysis of 10A isolates, we obtained publicly available genomic data from the European Nucleotide Archive.

### Statistical analysis

*S. pneumoniae* isolates were grouped into two vaccine periods based on the date of sample collection: the pre-PCV13 period (1997–2010) and the post-PCV13 period (2011–2023) [15,34]. Differences in the distribution of serotypes, GPSCs and resistance to each antibiotic between PCV13 groups or disease groups were assessed using Fisher’s exact test, along with the odds ratio (OR) and 95% confidence interval (CI). All tests were two-tailed, and *P* value<0.05 was considered statistically significant. All statistical analyses were performed using SPSS software (IBM Corp., Armonk, NY).

## Results

### Collection of pneumococcal isolates

A total of 236 pneumococcal isolates were collected and analysed, with 13.1% (31/236) collected during the pre-PCV13 period (1997–2010) and 86.9% (205/236) during the post-PCV13 period (2011–2023) (Table S1). Of these, 26.7% (63/236) were derived from IPD, with the majority (98.4%, 62/63) isolated from blood. High-quality whole-genome sequences were obtained from all isolates, with an average N50 value of 93.4 Kb (range, 38.0–266.7 Kb).

### Serotype distribution

The concordance rate of serotyping between multiplex PCR and WGS was 79.7% (126/158) (Table S1), which is consistent with a previous study [35]. A total of 37 serotypes were identified among the 236 pneumococcal isolates (Table S1). The most prevalent serotype was 23A (27/236, 11.4%), followed by 11A (23/236, 9.7%), 19A (21/236, 8.9%), 15B/15C (20/236, 8.5%), 34 (14/236, 5.9%) and 35B (14/236, 5.9%) (Table 1).

**Table 1.** Serotype distribution of pneumococcal isolates by vaccine period and disease

Serotype*(*N*)	Vaccine introduction period			Disease		
	Pre-PCV13(*N*=31)	Post-PCV13(*N*=205)	*P* value	Non-IPD(*N*=173)	IPD(*N*=63)	*P* value
1* (2)	2 (6.5%)	0	0.017	1 (0.6%)	1 (1.6%)	0.463
3* (3)	0	3 (1.5%)	1.000	2 (1.2%)	1 (1.6%)	1.000
4* (1)	1 (3.2%)	0	0.131	0	1 (1.6%)	0.267
5* (1)	1 (3.2%)	0	0.131	0	1 (1.6%)	0.267
6A* (10)	2 (6.5%)	8 (3.9%)	0.624	8 (4.6%)	2 (3.2%)	1.000
6B* (1)	0	1 (0.5%)	1.000	1 (0.6%)	0	1.000
6C (10)	0	10 (4.9%)	0.367	9 (5.2%)	1 (1.6%)	0.297
6D (4)	2 (6.5%)	2 (1.0%)	0.085	3 (1.7%)	1 (1.6%)	1.000
6E (2)	1 (3.2%)	1 (0.5%)	0.246	1 (0.6%)	1 (1.6%)	0.463
7C (1)	0	1 (0.5%)	1.000	0	1 (1.6%)	0.267
7F* (2)	2 (6.5%)	0	0.017	1 (0.6%)	1 (1.6%)	0.463
8 (3)	0	3 (1.5%)	1.000	1 (0.6%)	2 (3.2%)	0.175
9N (3)	0	3 (1.5%)	1.000	0	3 (4.8%)	0.018
9V* (3)	2 (6.5%)	1 (0.5%)	0.046	1 (0.6%)	2 (3.2%)	0.175
10A (8)	0	8 (3.9%)	0.601	3 (1.7%)	5 (7.9%)	0.033
11A (23)	2 (6.5%)	21 (10.2%)	0.747	19 (11.0%)	4 (6.3%)	0.334
11E (2)	0	2 (1.0%)	1.000	2 (1.2%)	0	1.000
12F (3)	1 (3.2%)	2 (1.0%)	0.346	0	3 (4.8%)	0.018
13 (4)	1 (3.2%)	3 (1.5%)	0.433	3 (1.7%)	1 (1.6%)	1.000
14* (1)	0	1 (0.5%)	1.000	0	1 (1.6%)	0.267
15A (4)	0	4 (2.0%)	1.000	3 (1.7%)	1 (1.6%)	1.000
15B/15C (20)	0	20 (9.8%)	0.084	17 (9.8%)	3 (4.8%)	0.294
16F (1)	0	1 (0.5%)	1.000	1 (0.6%)	0	1.000
17A (2)	0	2 (1.0%)	1.000	2 (1.2%)	0	1.000
17F (3)	1 (3.2%)	2 (1.0%)	0.346	1 (0.6%)	2 (3.2%)	0.175
18C* (1)	0	1 (0.5%)	1.000	1 (0.6%)	0	1.000
19A* (21)	4 (12.9%)	17 (8.3%)	0.494	19 (11.0%)	2 (3.2%)	0.072
19F* (10)	3 (9.7%)	7 (3.4%)	0.130	8 (4.6%)	2 (3.2%)	1.000
20 (5)	1 (3.2%)	4 (2.0%)	0.509	3 (1.7%)	2 (3.2%)	0.612
22F (10)	0	10 (4.9%)	0.367	7 (4.0%)	3 (4.8%)	0.729
23A (27)	0	27 (13.2%)	0.031	27 (15.6%)	0	<0.001
23B (3)	0	3 (1.5%)	1.000	2 (1.2%)	1 (1.6%)	1.000
23F* (11)	2 (6.5%)	9 (4.4%)	0.642	6 (3.5%)	5 (7.9%)	0.168
33F (2)	0	2 (1.0%)	1.000	1 (0.6%)	1 (1.6%)	0.463
34 (14)	1 (3.2%)	13 (6.3%)	0.701	8 (4.6%)	6 (9.5%)	0.210
35B (14)	2 (6.5%)	12 (5.9%)	1.000	11 (6.4%)	3 (4.8%)	0.765
35D (1)	0	1 (0.5%)	1.000	1 (0.6%)	0	1.000

*PCV13 VTs.

As expected, PCV13-VTs, including serotype 1 (2/31 vs 0/205; *P*=0.017; OR=0.94, 95% CI: 0.85–1.03), 7F (2/31 vs 0/205; *P*=0.017; OR=0.94, 95% CI: 0.85–1.03) and 9V (2/31 vs 1/205; *P*=0.046; OR=0.07, 95% CI: 0.01–0.81), were significantly more frequent during the pre-PCV13 period. In contrast, NVT 23A (0/31 vs 27/205; *P*=0.031; OR=1.15, 95% CI: 1.09–1.22) was significantly more frequent during the post-PCV13 period (Table 1).

Additionally, five NVTs with more than five isolates were exclusively identified in the post-PCV13 period (Table 1): 23A (27/205, 13.2%), 15B/15C (20/205, 9.8%), 6C (10/205, 4.9%), 22F (10/205, 4.9%) and 10A (8/205, 3.9%). The serotype coverage of PCV13 was 28.4% (67/236) overall, 61.3% (19/31) in the pre-PCV13 period and 23.4% (48/205) in the post-PCV13 period. The theoretical coverage of the FDA-approved PCV20 serotype (adding 8, 10A, 11A, 12F, 15B, 22F and 33F) increased to 57.6% (136/236) overall, underscoring its potential in further reducing IPD caused by non-PCV13 serotypes [36].

From a disease invasiveness perspective, the most common serotypes among the 63 IPD isolates were 34 (6/63, 9.5%), 10A (5/63, 7.9%), 23F (5/63, 7.9%) and 11A (4/63, 6.3%). Among the 173 non-IPD isolates, the most common serotypes were 23A (27/173, 15.6%), 11A (19/173, 11.0%), 19A (19/173, 11.0%), 15B/15C (17/173, 9.8%), 35B (11/173, 6.4%) and 6C (9/173, 5.2%) (Table 1). Serotypes 9N (0/173 vs 3/63; *P*=0.018; OR=1.05, 95% CI: 0.99–1.11), 10A (3/173 vs 5/63; *P*=0.033; OR=4.89, 95% CI: 1.13–21.08) and 12F (0/173 vs 3/63; *P*=0.018; OR=1.05, 95% CI: 0.99–1.11) were significantly more frequent among IPD isolates than among non-IPD isolates (Table 1). Notably, these three serotypes were all NVTs for PCV13, but PCV20 included serotypes 10A and 12F. In contrast, serotype 23A was exclusively detected in non-IPD isolates (27/173 vs 0/63; *P*<0.001; OR=0.84, 95% CI: 0.79–0.90). Among IPD isolates, serotype coverage was 30.2% (19/63) with PCV13, increasing to 63.5% (40/63) with PCV20.

### Pneumococcal lineages

**Fig. 1.**
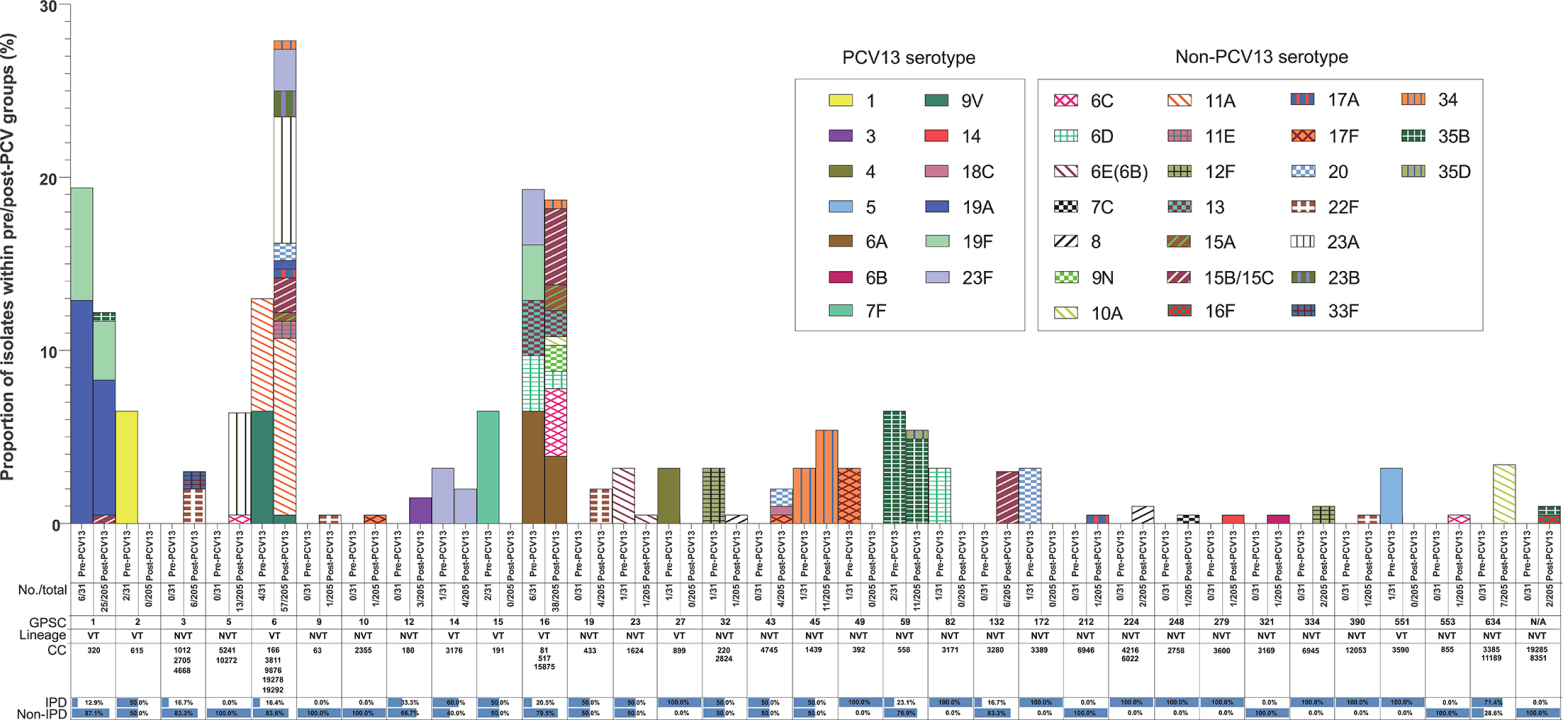
GPSC dynamics among pneumococcal strains isolated in South Korea, 1997–2023. The proportions of isolates during the pre-PCV13 and post-PCV13 periods are plotted by GPSC lineages across the vaccine periods. Isolates that could not be assigned to a GPSC are labelled as na. Serotypes included in PCV13 are shown with solid colour fills, whereas those included in non-PCV13 are shown with hatched pattern fills. A VT lineage was defined as having ≥50% VTs in the pre-PCV13 period, and an NVT lineage as having >50% NVTs in the pre-PCV13 period. Lineage-specific CCs and STs are shown underneath the graph. IPD proportions by lineage are indicated by blue horizontal bars. Blue and orange represent the percentage of isolates of each serotype in the pre-PCV13 and post-PCV13 periods, respectively.

A total of 32 GPSCs and 84 sequence types (STs) were identified among 236 pneumococcal isolates, including 8 newly assigned STs in the BIGSdb database (Table S1) [26]. These STs were further grouped into 46 CCs, with each CC assigned to only one GPSC (Fig. 1), reflecting a strong association between CCs and GPSCs [37]. The most prevalent GPSCs were GPSC6 (61/236, 25.8%; mainly CC166, serotypes 11A/23A), GPSC16 [44/236, 18.6%; mainly CC81, serotypes 6A/6C/(15B/15C)] and GPSC1 [31/236, 13.1%; CC320, serotypes (15B/15C)/19A/19F/35B], together accounting for 57.6% of the isolates. These lineages were predominant in South Korea, regardless of PCV13 introduction or disease invasiveness (Fig. 1 and Table S2).

Consistent with the serotype distribution, GPSC2 (CC615, serotype 1; *P*=0.017; OR=0.94, 95% CI: 0.85–1.03) and GPSC15 (CC191, serotype 7F; *P*=0.017; OR=0.94, 95% CI: 0.85–1.03) were significantly more frequent in the pre-PCV13 period (Table S2). Regarding invasiveness, GPSC634 (mainly CC11189, serotype 10A; *P*=0.016; OR=7.37, 95% CI: 1.39–39.02) was significantly associated with IPD isolates, whereas GPSC5 (mainly CC10272, serotype 23A; *P*=0.023; OR=0.93, 95% CI: 0.89–0.97) and GPSC6 (mainly CC166, serotypes 11A/23A; *P*=0.043; OR=0.45, 95% CI: 0.21–0.96) were more common in non-IPD isolates (Table S2).

### Capsular switching events

**Fig. 2.**
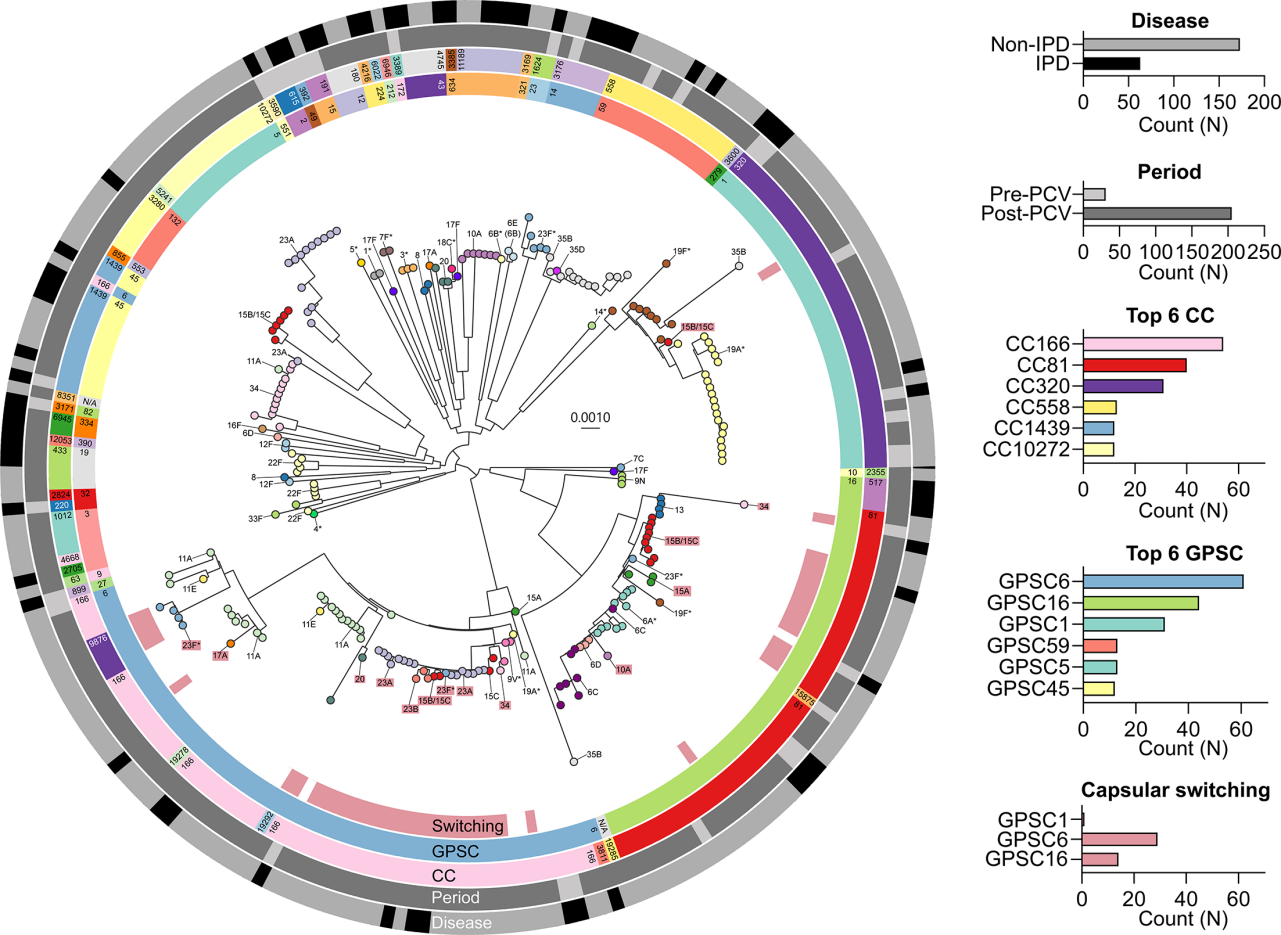
Maximum-likelihood tree and serotype distribution of 236 pneumococcal isolates collected in South Korea between 1997 and 2023. The tree was constructed based on core gene SNPs and was midpoint-rooted. Tree tip colours represent the serotypes. The colour strips represent the potential capsular switching (Switching), GPSC, CC, vaccine periods (Period) and disease group (Disease) from the inside out. The bar chart indicates the count of isolates by Disease, Period, CC, GPSC and Switching, according to the colour code in the colour strips.

Three GPSCs (1, 6 and 16) exhibited 44 potential capsular switching events (Fig. 2). GPSC1 expressed either serotype 19F (VT) or 19A (VT) during the pre-PCV13 period and subsequently expressed an additional 15B (NVT) during the post-PCV13 period (Fig. 2). Phylogenetic analysis suggested that the ancestral 19F-expressing clone evolved into 19A or 15B/15C variants (Fig. 2). Recombination events spanning the capsular polysaccharide synthesis (*cps*) locus (from *wzg* to *aliA*) were observed in GPSC1 (Fig. S1). GPSC6 and GPSC16 accounted for 29 (29/44, 65.9%) and 14 (14/44, 31.8%) of the total capsular switching events, respectively (Figs 2 and S1) Notably, 26 of the 44 switching events (59.1%) involved transitions from VT to NVT serotypes (Table S1).

### Antimicrobial resistance

Overall, the highest non-susceptibility rate was observed for azithromycin (187/226, 82.7%), followed by tetracycline (173/226, 76.5%), co-trimoxazole (159/226, 70.4%), clindamycin (140/226, 61.9%) and meropenem (112/226, 50.4%) (Table 2). Non-susceptibility rates for penicillin and cefotaxime were 19.5% (44/226) and 14.2% (32/226), respectively. Significantly higher non-susceptibility rates for amoxicillin (*P*=0.049; OR=3.00; 95% CI: 1.00–9.00) and meropenem (*P*=0.002; OR=4.23; 95% CI: 1.64–10.87) in the post-PCV13 period (1997–2010) than in the pre-PCV13 period (2011–2023) were observed, although a causal link to vaccine introduction could not be established. IPD isolates generally exhibited lower non-susceptibility across most antibiotics than non-IPD isolates (Table 2).

**Table 2.** Proportion of pneumococcal isolates with antibiotic non-susceptibility according to vaccine period or IPD

Antibiotic agent	Overall(*N*=226)	Agreement*	Vaccine introduction period			Disease		
			Pre-PCV13(*N*=28)	Post-PCV13(*N*=198)	*P* value	Non-IPD(*N*=166)	IPD(*N*=60)	*P* value
Penicillin	44 (19.5%)	74.2%	3 (10.7%)	41 (20.7%)	0.308	42 (25.3%)	2 (3.3%)	<0.001
Amoxicillin	70 (31.0%)	78.7%	4 (14.3%)	66 (33.3%)	0.049	62 (37.3%)	8 (13.3%)	0.001
Cefotaxime	32 (14.2%)	76.9%	3 (10.7%)	29 (14.6%)	0.775	28 (16.9%)	4 (6.7%)	0.054
Meropenem	112 (49.6%)	67.6%	6 (21.4%)	106 (53.5%)	0.002	90 (54.2%)	22 (36.7%)	0.024
Azithromycin	187 (82.7%)	97.8%	21 (75.0%)	166 (83.8%)	0.284	145 (87.3%)	42 (70.0%)	0.005
Clindamycin	140 (61.9%)	79.6%	15 (53.6%)	125 (63.1%)	0.406	107 (64.5%)	33 (55.0%)	0.216
Levofloxacin	28 (12.4%)	96.0%	5 (17.9%)	23 (11.6%)	0.359	25 (15.1%)	3 (5.0%)	0.042
Tetracycline	173 (76.5%)	93.4%	21 (75.0%)	152 (76.8%)	0.814	131 (78.9%)	42 (70.0%)	0.213
Co-trimoxazole	159 (70.4%)	95.1%	18 (64.3%)	141 (71.2%)	0.508	129 (77.7%)	30 (50.0%)	<0.001
Vancomycin	3 (1.3%)	–	0	3 (1.5%)	1.000	3 (1.8%)	0	0.567

*The agreement between AST and *in silico* prediction. *In silico* prediction for *β*-lactam was determined according to the PBP type.

Distinct lineage-specific patterns of antimicrobial non-susceptibility were observed (Table S3). Among the lineages, both GPSC1 and GPSC6 displayed significantly higher non-susceptibility to *β*-lactams (OR=3.22–6.26 for GPSC1; 3.96–10.19 for GPSC6), azithromycin and co-trimoxazole. Additionally, GPSC1 had increased non-susceptibility to tetracycline (OR=5.14; 95% CI: 1.18–22.29), while GPSC6 showed increased non-susceptibility to levofloxacin (OR=4.67; 95% CI: 2.06–10.60) and clindamycin (OR=8.37; 95% CI: 3.42–20.52) (Table S3).

Phenotypic AST results showed strong concordance with *in silico* predictions for azithromycin (221/226, 97.8%), levofloxacin (217/226, 96.0%), co-trimoxazole (215/226, 95.1%) and tetracycline (211/226, 93.4%) (Table S1). In contrast, concordance for *β*-lactams was relatively low: 74.2% (167/225) for penicillin and 78.7% (177/225) for amoxicillin. This suggests the need for reassessment and improvement of *β*-lactam resistance predictions based on PBP types in pneumococcal isolates from South Korea.

### Genomic characteristics of serotype 10A isolates

MLST analysis identified three distinct STs among the eight serotype 10A isolates (Table 3): ST11189 (*n*=6), ST3385 (*n*=1, tri-locus variant of ST11189) and ST12077 (*n*=1). The ST11189 and ST3385 isolates belonged to GPSC634, with 71.4% (5/7) derived from IPD cases, while the ST12077 isolate, assigned to GPSC16, was detected in a non-IPD case. The GPSC16 10A-ST12077 isolates exhibited evidence of capsular switching, while the other 10A isolates did not exhibit a capsular switching event (Fig. 2).

**Table 3.** Genomic characteristics of serotype 10A pneumococcal isolates

Strain	Disease	Serotype	ST	GPSC	MIC (µg ml^−1^)				Resistance gene		PBP type
					PEN	CLI	AZM	TET	*ermB*	*tetM*	
KSPN0117	Non-IPD	10A	12,077	16	1	0.5	4	8	No	Yes	15-12-18
KSPN0181	IPD	10A	3,385	634	0.125	0.06	0.125	0.06	No	No	146-new-7
KSPN0182	IPD	10A	11,189	634	2	64≤	64≤	32	Yes	Yes	15-89-18
KSPN0183	IPD	10A	11,189	634	2	na	64≤	32	Yes	Yes	15-89-18
KSPN0184	IPD	10A	11,189	634	0.25	64≤	64≤	32	Yes	Yes	15-89-18
KSPN0190	IPD	10A	11,189	634	1	64≤	64≤	32	Yes	Yes	15-89-18
KSPN0224	Non-IPD	10A	11,189	634	2	64≤	64≤	32	Yes	Yes	15-89-18
KSPN0242	Non-IPD	10A	11,189	634	2	1	16	32	Yes	Yes	15-89-18

na, not applicable.

AZM, azithromycin; CLI, clindamycin; PEN, penicillin; TET, tetracycline.

The minimum inhibitory concentrations (MICs) for penicillin, clindamycin, azithromycin and tetracycline in ST11189 isolates reached up to 2, ≥64, ≥64 and 32 µg ml^−1^, respectively. These values were notably higher than those observed in the ST3385 isolate within GPSC634 (Table 3). Additionally, all ST11189 genomes harboured the *ermB* (erythromycin and azithromycin resistance) and *tetM* (tetracycline resistance) genes, which were absent in the ST3385 genome.

**Fig. 3.**
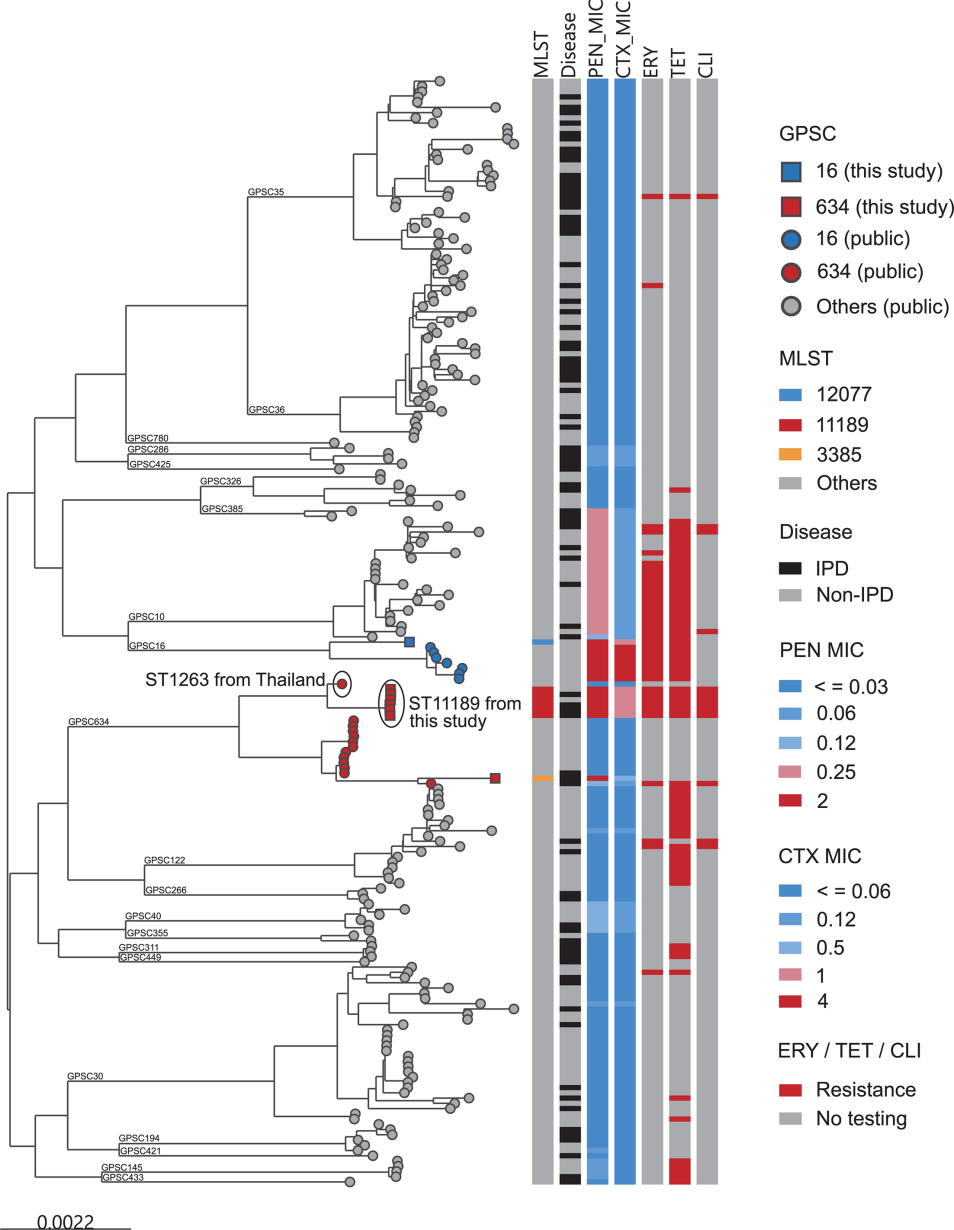
Maximum-likelihood tree of 211 serotype 10A pneumococcal genomes. The global serotype 10A pneumococcal genomes were downloaded from the European Nucleotide Archive. The tree was constructed based on core gene SNPs and can be interactively visualized at https://microreact.org/project/spn-southkorea-1997-2023-fig3. Tree tip colours represent the GPSC. The colour strips represent the MLST, disease group, MICs for penicillin and cefotaxime and resistance to erythromycin, tetracycline and clindamycin. PEN: penicillin, CTX: cefotaxime, ERY: erythromycin, TET: tetracycline, CLI: clindamycin.

Phylogenetic analysis further revealed that the GPSC634-ST11189 isolates shared a common ancestor with GPSC634-ST1263 (a double-locus variant of ST11189), identified in Thailand in 2007, and had undergone additional genetic changes since divergence (Fig. 3). Notably, *in silico* prediction indicated elevated MICs for penicillin (2 µg ml^−1^) and cefotaxime (1 µg ml^−1^) exclusively in ST11189 isolates compared to other STs within the same lineage.

### Virulence factors associated with invasiveness

A total of 38 virulence factors were identified across the 236 pneumococcal genomes, with an average of 27.3 factors per genome (range: 23–32) (Table S4). Among these, two genes, pneumococcal surface protein A (*pspA*; *P*=0.001; OR=8.24; 95% CI: 2.11–32.16) and plasmin- and fibronectin-binding protein A (*pfbA*; *P*=0.005; OR=3.72; 95% CI: 1.40–9.88), were significantly more frequent in IPD isolates. The *pspA* gene inhibits complement deposition, while *pfbA* facilitates host cell adherence. In contrast, pilus islet PI-1-related genes, including *rrgA*, *rrgB*, *rrgC*, *srtB* and *srtC*, which encode structural and sortase components of the surface pilus, were significantly associated with non-IPD isolates (*P*=0.019; OR=0.48; 95% CI: 0.27–0.88) (Table S5).

## Discussion

This study provides a comprehensive analysis of pneumococcal isolates collected in South Korea over 27 years, highlighting the evolving serotype distribution, AMR patterns and lineage-specific characteristics following the introduction of PCV13. Since the introduction of PCV13, serotype 10A has emerged as one of the predominant NVTs in IPD cases. These isolates were genetically related to a strain isolated in Thailand in 2007 and exhibited elevated MICs for *β*-lactams. WGS-based clustering with international isolates allowed the tracing of strain origins and determination of whether certain serotypes were independently introduced from abroad or originated domestically through capsular switching [38]. In this study, ST11189-10A showed no evidence of capsular switching and clustered closely with the Thailand isolate but had undergone further genetic changes compared to the Thai isolate. These genomic findings, combined with our epidemiological results, have important implications for pneumococcal disease prevention strategies in the era of vaccines with expanded serotype coverage.

The significant shift in serotype prevalence before and after the introduction of PCV13 demonstrates the well-documented phenomenon of serotype replacement [5]. Following the reduction in VTs, such as serotypes 1, 7F and 9V, NVTs such as 23A, 15B/15C and 10A have emerged as prominent contributors to the disease burden. This aligns with previous studies reporting similar trends globally, underscoring the dynamic nature of pneumococcal epidemiology in response to vaccine pressure [39]. An intriguing observation in our study is the differential distribution of serotypes between IPDs and non-IPDs. Specifically, serotype 23A was significantly more frequent in non-IPDs, whereas serotype 10A predominated among IPDs, consistent with trends in other countries [16,40,42]. The inclusion of serotype 10A in PCV20 suggests the potential for further reductions in IPD incidence with this newer vaccine formulation [43].

The identification of 32 GPSCs underscores the genetic diversity of pneumococcal populations. The level of diversity was comparable to that reported in other studies with similar sample sizes conducted in other countries [44,46]. This diversity is a hallmark of pneumococcal adaptability, driven by its capacity to colonize diverse hosts, evade immune responses and acquire genetic material through horizontal gene transfer. The predominance of GPSC6, GPSC16 and GPSC1, which collectively account for over half of the isolates in this study, aligns with global trends reporting the persistence of dominant lineages despite vaccine-induced selection pressures [47]. The detection of 44 capsular switching events, particularly between VTs and NVTs, emphasizes the adaptive capacity of pneumococci to evade vaccine-induced immunity. Such events highlight the need for genomic surveillance to track the evolution of vaccine escape variants [48].

Virulence factor analysis revealed significant associations between *pspA* and *pfbA* genes and IPDs, consistent with their known roles in immune evasion and host cell adhesion [49,50]. Conversely, pilus islet PI-1-related genes were more frequently detected in non-IPDs, suggesting their potential role in colonization rather than invasive disease.

Our findings highlight significant patterns of antimicrobial non-susceptibility in *S. pneumoniae* isolates, with azithromycin showing the highest non-susceptibility rate, followed by tetracycline, co-trimoxazole and *β*-lactams. Notably, while non-susceptibility rates for amoxicillin and meropenem were significantly higher in the post-PCV13 period, no direct causal relationship with vaccine introduction was identified. Distinct lineage-specific non-susceptibility patterns were observed, with GPSC1 and GPSC6 showing greater resistance to azithromycin and co-trimoxazole. In South Korea, a high proportion of pneumococcal isolates harbour the *ermB* gene, conferring macrolide resistance, with a reported genotype-phenotype concordance rate of 97.8%. Data from the Asian Network for Surveillance of Resistant Pathogens study, which collected isolates between 1998 and 2001, showed that 59.3% of *S. pneumoniae* isolates exhibited macrolide resistance in this region [51]. Given the historically high prevalence of macrolide resistance, azithromycin is not recommended as a first-line therapy for pneumococcal infections in South Korea. Molecular epidemiological studies suggest that the widespread presence of macrolide resistance is largely due to the horizontal transfer of the *ermB* gene and the clonal spread of resistant strains. Therefore, the persistently high resistance rates are likely due to early and widespread genetic acquisition and dissemination events rather than due to recent antibiotic selection pressure alone.

*In silico* prediction based on the detection of resistance genes showed high concordance with AST results, whereas *β*-lactam resistance predictions based on PBP type were relatively less consistent. Previous studies have reported a 98.7–100% agreement rate for penicillin and cefotaxime [52,53], whereas a study in Pakistan reported a lower rate of 75.5% for penicillin [25]. Given the limited availability of pneumococcal genomes from South Korea, it is possible that the current PBP profile, which serves as the basis for *β*-lactam resistance prediction, does not sufficiently include PBP types specific to South Korea. In our dataset, among the 58 non-concordant isolates for penicillin, 12 (12/58, 20.7%) harboured new alleles in at least one PBP gene. Similarly, new PBP alleles were identified in 7/48 (14.6%) non-concordant isolates for amoxicillin, 9/52 (17.3%) for cefotaxime and 14/73 (19.2%) for meropenem. Further development of a resistance prediction model incorporating pneumococcal genomes from South Korea will help improve prediction accuracy.

Although this study provides valuable insights into the changing epidemiology of *S. pneumoniae* in South Korea, there are some limitations. First, the relatively small sample size may have led to an under- or overestimation of serotype replacement or IPD-associated genetic factors. In addition, the absence of clinical stratification, including age-related data, limited further analysis. Future studies with data on disease manifestation, along with a larger number of isolates, may help better understand pneumococcal population structures in South Korea.

In conclusion, this study highlights the dynamic epidemiology of pneumococcal populations in South Korea, emphasizing the impact of vaccine introduction on serotype replacement, AMR and genetic diversity. The emergence of NVTs such as serotype 10A and capsular switching events underscores the adaptability of pneumococci to selective pressures, reinforcing the need for genomic surveillance to monitor vaccine escape and resistance trends. As PCV20 is implemented, region-specific genomic data and larger studies incorporating clinical stratification will be essential for guiding effective prevention and treatment strategies.

## Supplementary material

10.1099/mgen.0.001433 Fig. S1.

10.1099/mgen.0.001433 Uncited Supplementary Material 1.

## Abbreviations

AMR: antimicrobial resistance
AST: antimicrobial susceptibility testing
CC: clonal complex
CI: confidence interval
GPSC: global pneumococcal sequence cluster
IPD: invasive pneumococcal disease
MIC: minimum inhibitory concentration
MLST: multi-locus sequence type
NVT: non-vaccine serotype
OR: odds ratio
PBP: penicillin-binding protein
PCV: pneumococcal conjugate vaccine
ST: sequence type
VT: vaccine serotype
WGS: whole-genome sequencing
