# Supplementary material for: Genomic analysis and pneumococcal population dynamics across PCV implementation in South Korea, 1997–2023
Source: Microb Genom. 2025 Jul 17;11(7):001433. doi: 10.1099/mgen.0.001433 (PMC12284405; doi:10.1099/mgen.0.001433)
Supplement: Fig. S1. [file mgen-11-01433-s001.pdf]

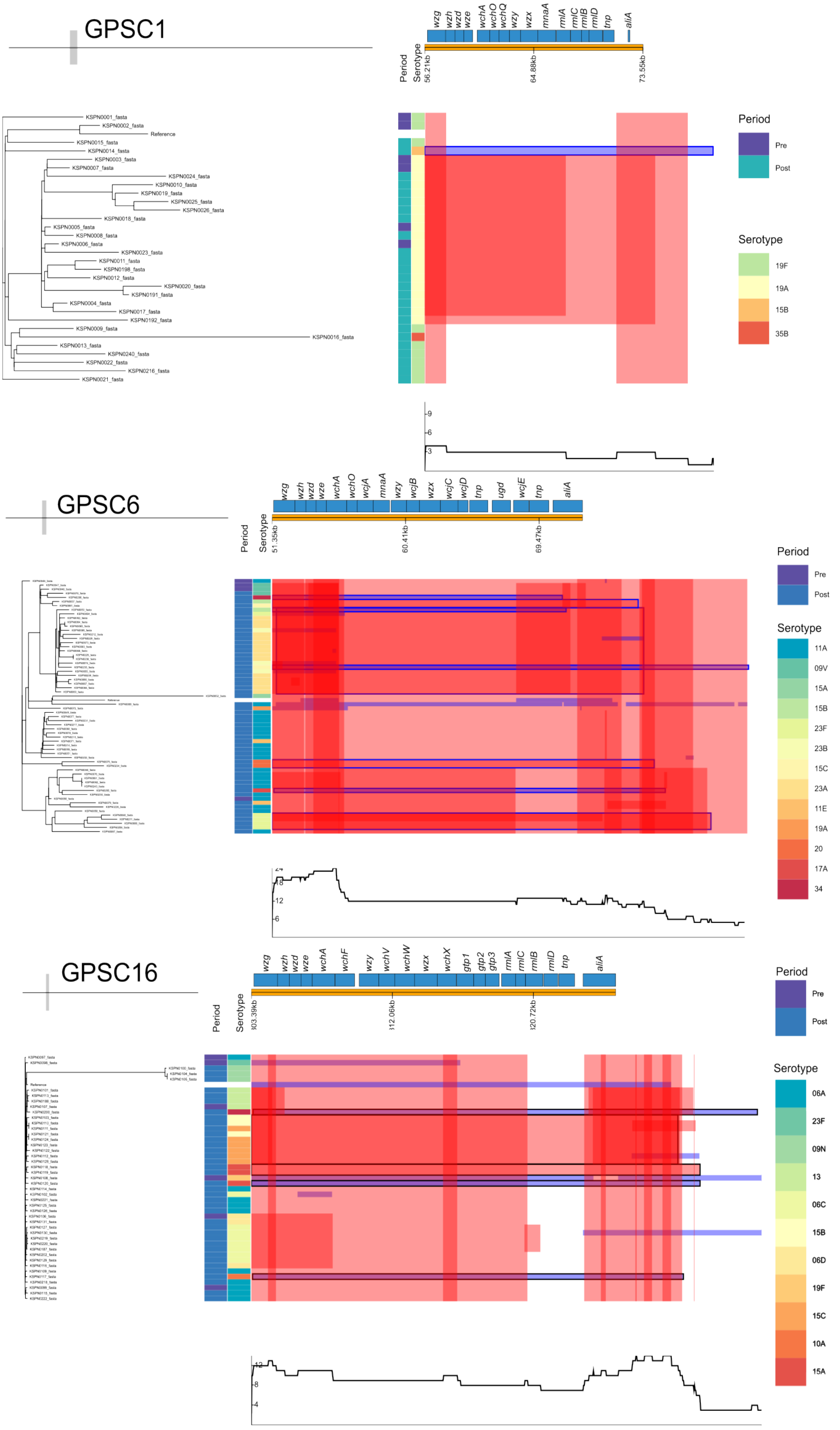

GPSC1
wzg
wzh
wzd
wze
wchA
wchO
wchQ
wzy
wzx
mnaA
rmlA
rmlC
rmlB
rmlD
tnp
aliA
Period
Serotype
56.21kb
64.88kb
73.55kb
KSPN0001_fasta
KSPN0002_fasta
Reference
KSPN0015_fasta
KSPN0014_fasta
KSPN0003_fasta
KSPN0007_fasta
KSPN0024_fasta
KSPN0010_fasta
KSPN0019_fasta
KSPN0025_fasta
KSPN0026_fasta
KSPN0018_fasta
KSPN0005_fasta
KSPN0008_fasta
KSPN0006_fasta
KSPN0023_fasta
KSPN0011_fasta
KSPN0198_fasta
KSPN0012_fasta
KSPN0020_fasta
KSPN0191_fasta
KSPN0004_fasta
KSPN0017_fasta
KSPN0192_fasta
KSPN0009_fasta
KSPN0016_fasta
KSPN0013_fasta
KSPN0240_fasta
KSPN0022_fasta
KSPN0216_fasta
KSPN0021_fasta
Period
Pre
Post
Serotype
19F
19A
15B
35B
9
6
3
GPSC6
wzg
wzh
wzd
wze
wchA
wchO
wcjA
mnaA
wzy
wcjB
wzx
wcjC
wcjD
tnp
ugd
wcjE
tnp
aliA
Period
Serotype
51.35kb
60.41kb
69.47kb
Period
Pre
Post
Serotype
11A
09V
15A
15B
23F
23B
15C
23A
11E
19A
20
17A
34
18
12
6
GPSC16
wzg
wzh
wzd
wze
wchA
wchF
wzy
wchV
wchW
wzx
wchX
gtp1
gtp2
gtp3
rmlA
rmlC
rmlB
rmlD
tnp
aliA
Period
Serotype
103.39kb
112.06kb
120.72kb
Period
Pre
Post
Serotype
06A
23F
09N
13
06C
15B
06D
19F
15C
10A
15A
12
8
4

Fig. S1. GPSC1, 6, and 16 capsular polysaccharide locus recombination events. Phandango plot of recombination detected with Gubbins, focused on the *cps* locus, across the phylogenies. Isolates are annotated with their PCV13 period and serotype in color strips and color legends specify the period and serotype information. Recombination blocks span recombination location of genes affected. Red blocks affect multiple isolates (n>1), blue blocks affect a single isolate on the tree tip (n=1). Overlapping blocks increase the density of the color. A sliding window of the number of recombination events affecting certain positions is plotted underneath. Among the emerged serotype(s) in post-PCV within a GPSC, the recombination block spanning the *cps* locus is outlined in blue.
